# Identification of Novel Regulators of Fruit Sugar Accumulation Based on Transcriptome and WGCNA in *Citrus sinensis*

**DOI:** 10.3390/ijms262412161

**Published:** 2025-12-18

**Authors:** Jianmei Chen, Chunli Xu, Zhenmin Chen, Qingyu Pei, Zixin Huang, Qiong Chen, Shubei Wan

**Affiliations:** National Navel Orange Engineering Research Center, College of Life Sciences, Gannan Normal University, Ganzhou 341000, China; chenjm@gnnu.edu.cn (J.C.); 1241016012@gnnu.edu.cn (C.X.); chen002529@163.com (Z.C.); 1231016003@gnnu.edu.cn (Q.P.); 1241016026@gnnu.edu.cn (Z.H.); qiong0552@163.com (Q.C.)

**Keywords:** citrus, sugar content, transcriptome analyses, WGCNA, key genes

## Abstract

Sweet orange (*Citrus sinensis*) is recognized as one of the most significant citrus fruits globally. The sugar content of fruits is the most critical internal quality associated with taste in sweet oranges, serving as a vital determinant of fruit quality and commercial value. Therefore, a comprehensive exploration of the regulatory mechanisms governing sugar accumulation during fruit ripening holds substantial value for high-quality fruit breeding. In this study, we investigated citrus sugar accumulation using the flesh of the Newhall navel orange and its high-sugar-content mutant cultivar, Ganmi, as experimental materials. RNA sequencing of the flesh from both Ganmi and Newhall oranges at 180 and 200 days after flowering identified 642 and 493 differentially expressed genes (DEGs), respectively. Functional enrichment analysis indicated that DEGs were mainly enriched in the sugar metabolism pathways, sugar transporters, and plant hormone signal transduction. Important DEGs associated with fruit sugar accumulation in Ganmi included *Cs_ont_2g004470* (*CsNAC73*) and *Cs_ont_9g005250* (*CsSTP13*) involved in sugar accumulation. Weighted gene co-expression network analysis showed that 20 co-expression modules were obtained, and the brown1 module had the strongest correlations with sugar content. Based on gene functionality and gene expression analyses of 1189 genes in this module, three genes (*Cs_ont_2g004470* (*CsNAC73*), *Cs_ont_5g050360* (*CsMYC2*) and *Cs_ont_3g002820* (*CsBBX21*)) were identified as key genes potentially related to sugar accumulation during the ripening. These findings may contribute to elucidating the mechanisms underlying sugar accumulation during ripening and provide insights for the molecular breeding of citrus varieties.

## 1. Introduction

Sugar content (measured as total soluble solids (TSS) in °Brix) is the most significant internal quality associated with the taste in fruits and is an important determinant of fruit quality and commercial value [1,2,3]. Therefore, fully exploring and utilizing the excellent regulatory genes for sugar accumulation during the ripening of fruits are of great value for high-quality fruit breeding.

Sugar accumulation and color change in fruits are common metrics for assessing ripeness. The ripening of fruit involves a complex process regulated by various factors at multiple levels [4,5,6,7]. One of the primary factors is hormones, particularly abscisic acid (ABA) and ethylene [3,8,9]. It is well established that ethylene serves as a key regulator of ripening in climacteric fruits, while ABA is believed to play a significant role in the ripening of non-climacteric fruits [10,11]. ABA has been shown to be crucial in the ripening process of non-climacteric fleshy fruits, such as citrus fruits, by modulating sugar accumulation [3,9,12]. However, the mechanisms through which ABA regulates sugar accumulation in non-climacteric fruits remain inadequately understood, though they are known to be complex. Therefore, it is urgent to accurately and effectively identify more sugar accumulation-related genes in non-climacteric fruits to enhance fruit taste.

A complex gene regulatory network is implicated in determining sugar transport, which plays a crucial role in the molecular mechanisms controlling sugar content in fruits [13]. Several genes, including those encoding transcription factors and functional proteins, are involved in the sugar accumulation process in plants. These functional proteins primarily consist of metabolic degradation enzymes and sugar transporters, including SUT (sucrose transporter), MST (monosaccharide transporter), TST (tonoplast sugar transporter), and SWEET (sugars will eventually be exported transporters; sugar effluxers) [14,15,16,17]. Furthermore, other functional proteins can regulate metabolic degradation enzymes and sugar transport proteins, thereby influencing fruit sugar accumulation [17,18]. In tomato (*Solanum lycopersicum*), calcium-dependent protein kinase 27 (SlCDPK27) and its paralogue SlCDPK26 control fruit sugar content by phosphorylating a sucrose synthase, which promotes degradation of the sucrose synthase [18]. Several transcription factors (TFs) that regulate the sugar content in fruits have been identified and characterized. Basic helix–loop–helix (bHLH) transcription factor CsbHLH122 physically interacts with MYB transcription factor CsMYBS3 to form a transcription factor complex, enhancing promoter transcriptional activity of sucrose transporter CsSUT2, thus positively regulating fruit sugar accumulation in citrus [19]. Basic leucine zipper (b-ZIP) transcription factor PbrbZIP15 induced soluble sugar accumulation during pear fruit development is at least partly attributed to the activation of glucose isomerase 1 gene (PbrXylA1) [20]. NAC (NAM, ATAF, and CUC) transcription factors VvNAC72 is an activator that binds to the VvNAC72-binding domain (CACATG) and promotes sugar transporter gene VvSWEET15 expression and hexose accumulation at postvéraison stages in grape [21].

Navel orange (*Citrus sinensis* Osbeck) is among the most significant citrus fruits globally and is extensively cultivated in Ganzhou, Jiangxi Province, China. In our previous studies, we reported a bud mutation characterized by enlarged organ size, designated M25 (Ganmi), which exhibits superior fruit quality and was isolated from the Newhall navel (NHE) orange [22]. In this study, we investigated the citrus sugar content of both the Newhall navel orange and its high-sugar mutant cultivar, Ganmi. We assessed genome-wide gene transcription levels at two sugar accumulation stages of fruit to elucidate the molecular mechanisms underlying sugar content in Ganmi. Additionally, we conducted a weighted gene co-expression network analysis (WGCNA) to identify hub genes. Our findings may elucidate the mechanisms influencing fruit sugar content and provide insights for the molecular breeding of citrus varieties.

## 2. Results

### 2.1. Fruit Morphology and Sugar Content in the Ganmi Navel Orange

**Figure 1:**
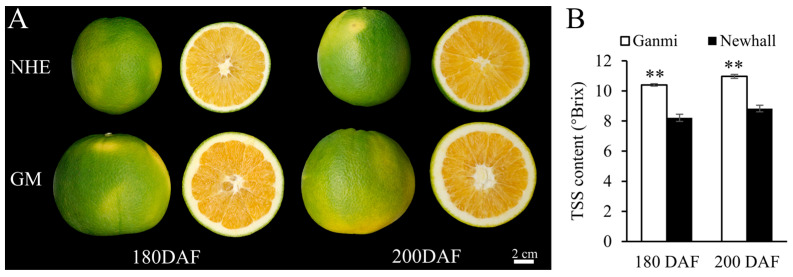
Comparison of fruit morphology and TSS content between Ganmi and Newhall navel orange: (**A**) Fruit morphology of Ganmi (GM) and Newhall (NHE) navel orange at 180 and 200 days after flower (DAF), scale bars = 2 cm; (**B**) TSS of fruit pulps of GM and NHE navel orange at 180 and 200 DAF. ** denote significant difference at the 0.01 probability levels.

No differences were observed in the onset of color variation between Ganmi and Newhall (Figure 1A). However, at 180 and 200 days after flowering (DAF), the TSS content of Ganmi navel oranges was significantly greater than that of Newhall navel oranges (Figure 1B). Following maturation, the total soluble solids (TSSs) of the Ganmi variety of navel orange were significantly higher than those of the wild Newhall variety (Figure S1). These findings indicate that the Ganmi navel orange has a higher sugar content during fruit ripening compared to the Newhall variety.

### 2.2. Analysis of Differentially Expressed Genes (DEGs) in the Pulps of Ganmi and Newhall Navel Oranges

**Figure 2:**
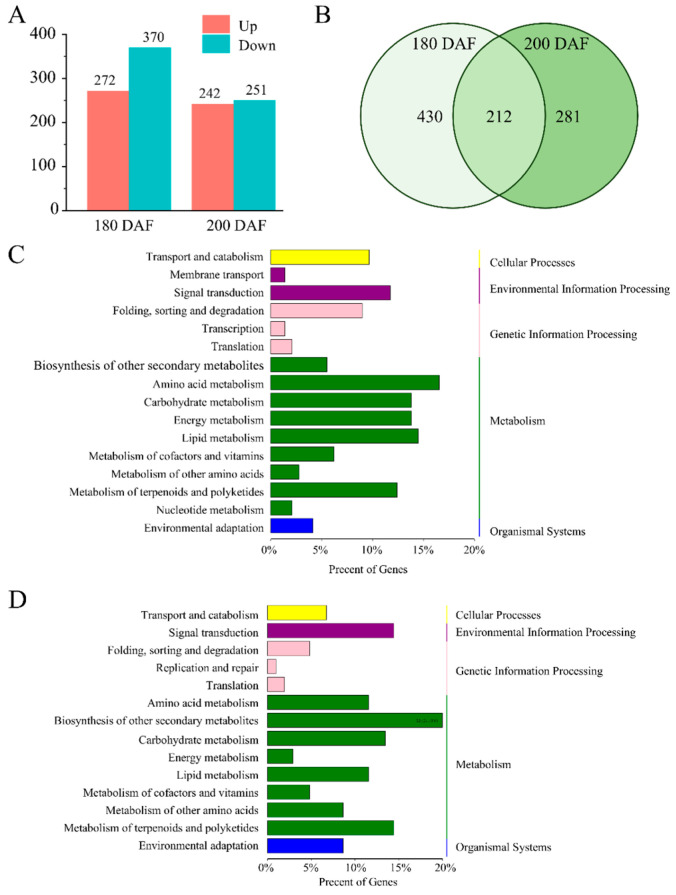
Identification and pathway enrichment analysis of DEGs related to fruit sugar accumulation in Ganmi: (**A**) The DEG numbers of the two groups. (**B**) Venn diagram analysis showing the number of common and unique DEGs identified at two stages. (**C**,**D**) KEGG enrichment pathway (level 1 and 2) annotated classification results of all DEGs in Ganmi and Newhall navel orange at 180, and 200 DAF, respectively.

Pulp samples from Ganmi and Newhall navel oranges at 180 and 200 days after flowering (DAF) were subjected to RNA sequencing (RNA-seq) analysis to examine the differentially expressed genes (DEGs) between the two cultivars throughout the accumulation of fruit sugars. DEGs were identified based on a false discovery rate (FDR) of <0.05 and |log2(fold change [FC])| ≥ 1. Using these criteria, we identified 642 DEGs at 180 DAF (272 upregulated and 370 downregulated) and 493 DEGs at 200 DAF (242 upregulated and 251 downregulated) (Figure 2A; Table S1; Figure S2). Among these DEGs, 212 were found to be common to both 180 and 200 DAF (Figure 2B; Table S1). Notably, the expression trends of these 212 genes were entirely consistent between 180 and 200 DAF (Table S2).

The Kyoto Encyclopedia of Genes and Genomes (KEGG) pathway analysis was performed to identify metabolic and genetic information processing pathways enriched in all differentially expressed genes (DEGs) (Figure 2C,D; Tables S3 and S4). The identified processes were classified into five main categories: metabolism, genetic information processing, environmental information processing, cellular processes, and organismal systems (Figure 2C,D). Among these, metabolism was the most enriched KEGG pathway across all samples. Specifically, there were 20 and 14 DEGs enriched in the ‘Carbohydrate metabolism’ pathways, including the ‘Glycolysis/Gluconeogenesis’, ‘Fructose and mannose metabolism’, ‘Glyoxylate and dicarboxylate metabolism’, ‘Citrate cycle’, and ‘Starch and sucrose metabolism’ pathways at 180 and 200 days after flowering (DAF), respectively. These results indicate that alterations in sugar metabolism occurred in the fruits of Ganmi navel oranges. Additionally, certain plant hormones influence sugar accumulation in fruits [6,23]. There were 10 DEGs enriched in the ‘Plant hormone signal transduction’ pathway at both 180 and 200 DAF. Functional annotation of the DEGs identified in this pathway revealed 5 and 4 DEGs related to auxin signaling transduction at 180 and 200 DAF, respectively.

A Gene Ontology (GO) enrichment analysis was conducted to classify the functions of all differentially expressed genes (DEGs) into three categories: biological processes, cellular components, and molecular functions. The identified 20 GO biological process terms demonstrated the greatest enrichment in both developmental stages (Figure S3A,B; Tables S5 and S6). At 180 days after flowering (DAF), DEGs in fruits were significantly enriched in processes related to sugar metabolism regulation, including the glycerol metabolic process (q = 1.562 × 10^−2^) and sucrose-mediated signaling (q = 6.021 × 10^−3^) (Figure S3A). At 200 DAF, DEGs in fruits were also enriched in processes regulating sugar transport, such as monosaccharide transmembrane transport and glucose import (Figure S3B). Additionally, seven DEGs in fruits at 180 DAF were significantly enriched in the abscisic acid biosynthetic process (q = 1.494 × 10^−3^), while five DEGs at 200 DAF were significantly enriched in the auxin biosynthetic pathway (q = 1.94 × 10^−2^) (Figure S3A,B). These results align with those obtained from the KEGG enrichment analysis.

**Figure 3:**
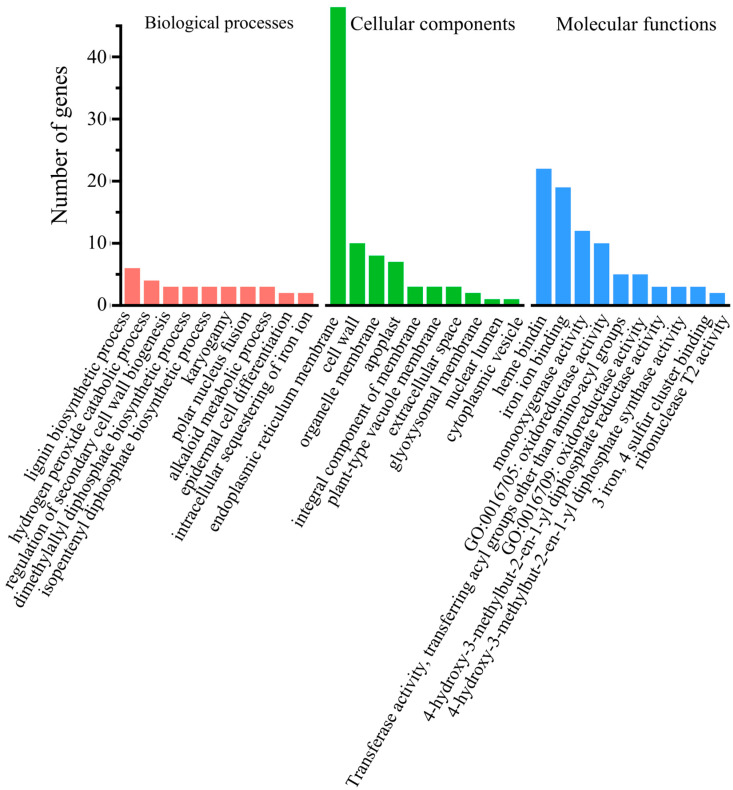
Gene Ontology (GO) classification of the DEGs, found to be common to both 180 and 200 DAF. Red indicates GO terms related to biological processes, green indicates GO terms related to cell components, and blue indicates GO terms related to molecular function.

To identify differences in gene expression between two cultivars, we focused on DEGs, which were found to be common to both 180 and 200 DAF. Among these 212 DEGs, some genes are known to be involved in fruit sugar accumulation. NAC73 is a known transcription factor that regulates sugar content in fruits. In strawberries, the NAC73 homolog FvNAC073 positively regulates the expression of the sucrose synthesis-related gene sucrose-6-phosphate synthase 1 (FvSPS1) by directly binding to its promoter while negatively regulating the expression of the sucrose degradation-related gene sucrose synthase 2 (FvSUS2), thereby positively influencing sugar accumulation in fruits [24]. In peach research, the NAC73 homolog PpNAC73 regulates the accumulation of sucrose in fruits by directly regulating the expression of PpbZIP18 and indirectly activating the expression of sucrose synthase 1 (PpSuSy1) and sucrose transport 1 (PpST1) [25]. NAC73 protein-encoding gene *Cs_ont_2g004470* was significantly upregulated in fruits of Ganmi at 180 and 200 DAF (Table S1). Furthermore. GO enrichment analysis of 212 differentially expressed genes, which were found to be common to both 180 and 200 DAF, was performed. The 48 (22.64%) DEGs were strongly enriched in the GO category ‘integral component of membrane’ (q = 6.397 × 10^−4^) (Figure 3). This indicates an altered membrane structure in the Ganmi navel oranges compared to Newhall navel oranges. Among the DEGs, the expression of sugar transporter gene *Cs_ont_9g005250*, which encodes sugar transport protein 13 (STP13), was upregulated in the fruits of Ganmi plants both at 180 and 200 DAF (Table S1). The STP13-mediated hexose uptake was identified as sugar transporters responsible for fruit sugar accumulation in strawberries [26].

### 2.3. Co-Expression Network Analysis

**Figure 4:**
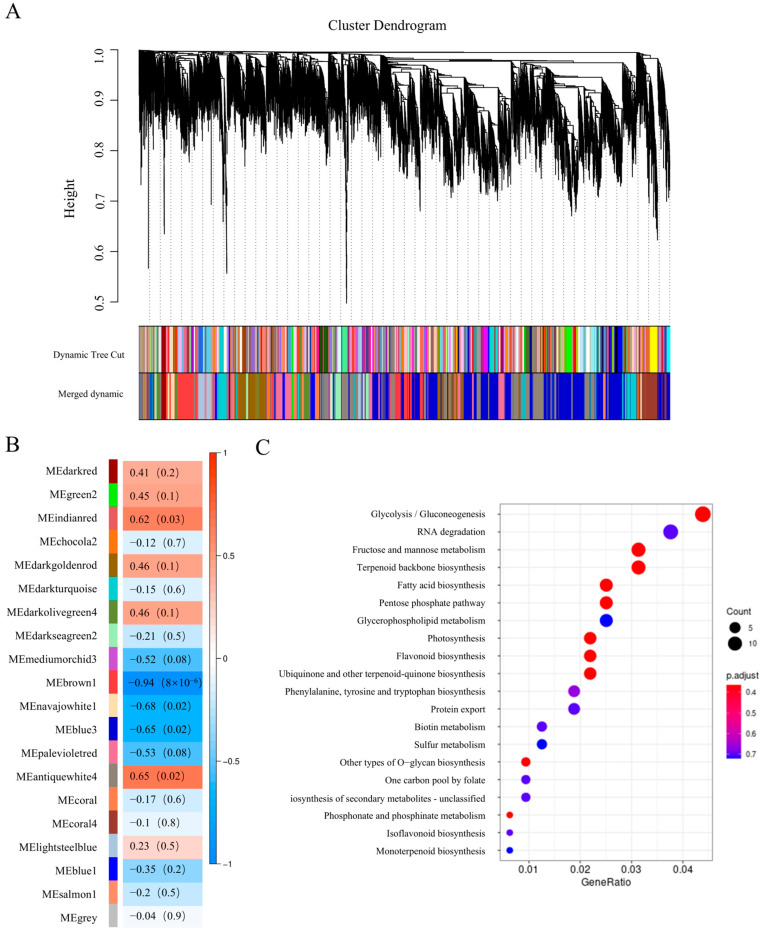
Identification of key regulators related to fruit sugar accumulation in Ganmi: (**A**) Weighted gene coexpression network analysis identifies 22 co-expression modules. (**B**) Module–trait relationships plot. The module sample correlation and corresponding *p*-values are shown in parentheses. The panel on the left shows 22 modules. The color code on the right shows the module feature correlation −1 (blue) to 1 (red); (**C**) KEGG enrichment analysis of genes in the brown1 module.

Co-expression network analysis was conducted to further investigate the transcriptional changes in two navel orange cultivars during fruit ripening. Genes exhibiting similar expression patterns were categorized into 20 modules, each assigned a distinct color (Figure 4A). Among these, the blue3 module contained the highest number of genes (4546), while the mediumorchid3 module had the lowest (25) (Figure S4). Analysis of module–trait relationships indicated that the brown1 module was significantly correlated with total soluble solids (TSS) content (r^2^ = −0.94, *p* = 4 × 10^−6^; Figure 4B). The brown1 module comprises 79 genes that are differentially expressed at both 180 and 200 days after flowering (DAF), including the NAC73 protein-encoding gene *Cs_ont_2g004470*. Furthermore, KEGG enrichment analysis of the genes within the brown1 module revealed that 10 and 14 genes are enriched in the ‘Fructose and mannose metabolism’ and ‘Glycolysis/Gluconeogenesis’ pathways, respectively (Figure 4C). These findings suggest that the brown1 module serves as a key regulator of high sugar content in Ganmi navel oranges.

### 2.4. Screening of Key Genes in Relevant Modules

A total of 1189 genes were included in the brown1 module. The lists of these genes and their annotations are presented in Table S7. The functional enrichment, expression patterns, and construction of an expression network for the genes in this module can help distinguish key regulators of sugar accumulation in navel oranges. Candidate transcription factors were screened based on Gene Significance (GS) > 0.8 and Module Membership (MM) > 0.8, resulting in the identification of 7 transcription factors (Table 1). Among these genes, several may be involved in sugar accumulation in fruit, as indicated by previous reports concerning other plants. The homologous gene *Cs_ont_2g004470* (*CsNAC73*) has been shown to participate in regulating fruit sugar accumulation in both climacteric and non-climacteric fruits [24,25]. *Cs_ont_2g025530* encodes an ethylene-responsive factor 113 (*CsERF113*), whose homologue mediates banana ripening by regulating starch and chlorophyll degradation [27]. Additionally, some genes involved in abscisic acid (ABA) signaling cannot be entirely excluded from the candidate gene list, despite not being directly associated with sugar accumulation. MYB1R1 and MYC2 are induced by ABA and bind to the PbFAD3a promoter to activate its expression, thereby regulating the color of the fruit peel [28]. The wheat heat shock factor genes *TaHsfA2h* and *HsfC2a* enhance heat tolerance under thermal stress by positively regulating ABA signaling [29]. AtBBX21 is a B-box zinc finger transcription factor that integrates light and ABA signals to regulate the elongation of the hypocotyl in *Arabidopsis thaliana* [30,31,32]. Therefore, the following genes were selected as candidate key regulators of sugar accumulation in the fruits of Ganmi navel oranges: *Cs_ont_2g004470* (*CsNAC73*), *Cs_ont_2g025530* (*CsERF113*), *Cs_ont_7g025420* (*CsMYB1R1*), *Cs_ont_5g050360* (*CsMYC2*), *Cs_ont_3g002820* (*CsBBX21*), and two heat stress transcription factor genes (*Cs_ont_2g014800* and *Cs_ont_9g006370*).

**Figure 5:**
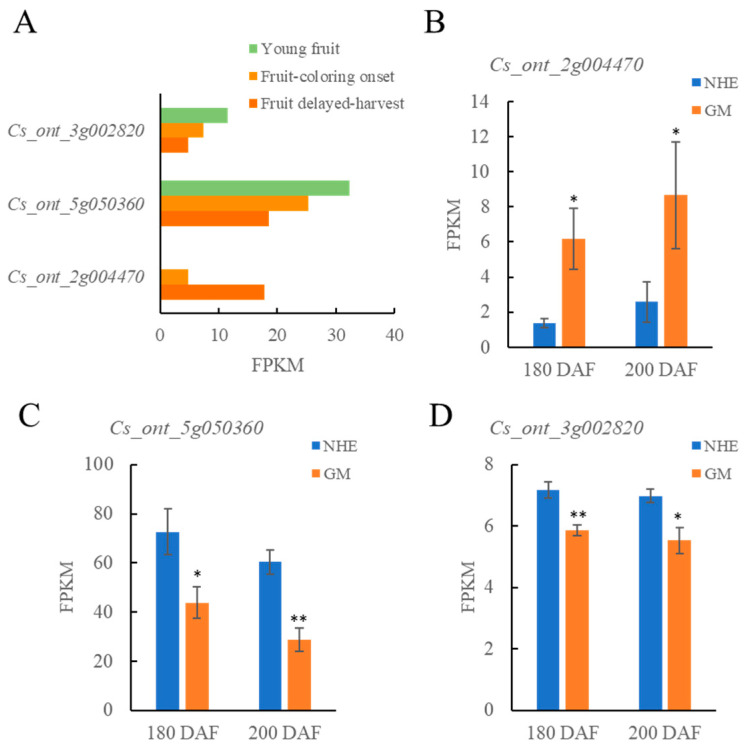
Gene expression profiling analysis of three key genes related to fruit sugar accumulation in Ganmi: (**A**) Expression characterization of three genes in fruits at different developmental stages. (**B**–**D**) Expression analysis of 3 sugar-accumulating related genes, *Cs_ont_2g004470* (**B**), *Cs_ont_5g050360* (**C**) and *Cs_ont_3g002820* (**D**), in fruits at 180 and 200 DAF. * and ** denote significant differences at the 0.05 and 0.01 probability levels, respectively.

The expressions of seven genes at different stages of fruit ripening were analyzed using transcriptomic data from previous studies (Table S8) [33]. *Cs_ont_2g004470* (*CsNAC73*), *Cs_ont_5g050360* (*CsMYC2*), and *Cs_ont_3g002820* (*CsBBX21*) were identified as key genes responsible for sugar accumulation in fruits based on gene expression profiling and functional annotation. As the fruit developed and ripened, the expression level of *Cs_ont_2g004470* (*CsNAC73*) gradually decreased in the flesh of *C. sinensis* (Figure 5A). In Ganmi plants, which have a higher sugar content, the expression of *Cs_ont_2g004470* (*CsNAC73*) was significantly higher than in plants at both 180 and 200 DAF (Figure 5B). Additionally, the expression levels of *Cs_ont_5g050360* (*CsMYC2*) and *Cs_ont_3g002820* (*CsBBX21*) also gradually decreased in the flesh of *C. sinensis* as the fruit matured (Figure 5A). Notably, the expression levels of *Cs_ont_5g050360* (*CsMYC2*) and *Cs_ont_3g002820* (*CsBBX21*) were significantly lower in the flesh of Ganmi compared to Newhall at both 180 and 200 DAF (Figure 5C,D).

## 3. Discussion

Citrus are important horticultural crops, and soluble sugars are a crucial indicator of citrus fruit quality. The mechanism of rapid accumulation of sugars during the maturation of citrus fruits has received increasing attention. Several key genes regulating sugar accumulation in citrus fruits have been identified, and research on their regulatory mechanisms has been conducted, including *CsbHLH122*, *CsMYBS3*, *CsCIPK23*, *CitSAR*, *CitZAT5*, and *CitNAC47* [17,19,23,34]. These genes initially constructed a regulatory network for sugar content in citrus fruits; however, the regulatory mechanisms remain unclear and require further investigation. This study utilized the new navel orange variety Ganmi, which exhibits a higher fruit sugar content, as the material and employed transcriptomics and co-expression network analysis to identify key regulatory genes and pathways influencing sugar content in citrus fruits, thereby providing new insights into the regulatory network of sugar content in these fruits.

Citrus fruits are classified as typical non-climacteric fruits, and numerous studies have identified that sugar accumulation is regulated by abscisic acid (ABA). Research on strawberry fruits has shown that the sugar content regulatory gene *FaTCP7* is modulated by ABA [26]. Additionally, sugar metabolism-related genes, such as *Sucrose Synthase 3* (*VvSS3*) and *Sucrose Non-Fermenting*-1 (SNF1)-related *Protein Kinase* 1 (*VvSnRK1*), respond to ABA signals to regulate sugar accumulation in grape fruits during ripening [5]. In this study, we observed that the differentially expressed genes (DEGs) between Ganmi and Navel oranges during fruit ripening were significantly enriched in hormone signal transduction pathways. Specifically, 10 and 12 genes were enriched in the Gene Ontology (GO) term ‘response to abscisic acid’ at 180 and 200 days, respectively. Furthermore, 7 DEGs in fruits at 180 days were significantly enriched in the GO term ‘abscisic acid biosynthetic process’. These findings indicate that ABA plays a crucial role in regulating the higher sugar content of Ganmi fruits.

We identified several differentially expressed genes (DEGs) related to auxin signaling transduction, such as *Cs_ont_5g011260*, which encodes the auxin-induced protein AUX28. This gene was downregulated in Ganmi compared to Newhall at both 180 and 200 days after flowering (DAF). Additionally, in this study, *Cs_ont_3g002820* (*CsBBX21*) was identified as a key gene regulating sugar accumulation. The homologous gene of *CsBBX21* inhibits the expression of auxin-related genes, including *SAUR24*, *SAUR29*, and *IAA29*. Currently, only a limited number of genes mediating auxin signal transduction to regulate sugar accumulation during fruit ripening have been identified [35]. Overall, auxin may affect sugar accumulation in the fruits of Ganmi.

Some studies have also indicated that auxin metabolism is influenced by ABA [36,37,38]. The interaction mechanism between auxins and ABA during fruit maturation is complex. ABA can regulate the expression of ARF (Auxin Response Factor) and Aux/IAA (Auxin/Indole-3-Acetic Acid protein) within the auxin signaling pathway at the transcriptional level [7]. These factors can, in turn, modulate the synthesis pathway or signaling process of ABA, thereby forming a complex regulatory network. Auxin and ABA may also play roles synergistically during the fruit development and ripening in Ganmi. This will require further experimentation.

## 4. Materials and Methods

### 4.1. Plant Materials

The Ganmi navel orange is a new variety that was originally isolated as a bud mutation from the Newhall navel orange in Ganzhou, China. Both Ganmi and Newhall navel oranges share the same genetic background. All plant materials were cultivated in the fields of the experimental nursery at Gannan Normal University and were planted under uniform conditions. Pulp samples for RNA-seq analysis were collected from Ganmi and Newhall plants at 180 and 200 days after flowering (DAF). Fruit samples were collected from three healthy trees, each 8 years old, in the field. Three fruits from each tree were dissected into small pieces and mixed uniformly to create one replicate. The samples were immediately frozen in liquid nitrogen and stored at −80 °C until further use.

### 4.2. Measurement of Flesh Soluble Solids Content

Pulp samples from Ganmi and Newhall were evaluated to determine the fruit soluble solid concentrations at 180 and 200 DAF. At each sampling time-point, three fruits were collected from each tree, and fruits from three trees were collected at each developmental stage as three biological replicate samples. The total soluble solid content (°Brix) of pulps was determined using digital hand-held refractometer (Atago Co., PAL-1, Tokyo, Japan) with an accuracy of 0.1 and calibrated using distilled water.

### 4.3. RNA Preparation

Total RNA was extracted from pulp tissues using the FastPure Universal Plant Total RNA Isolation Kit (Vazyme, Nanjing, China). RNA integrity and concentration were verified by Agilent 2100 bioanalyzer (Agilent, Santa Clara, CA, USA) and spectrophotometry (Nanodrop ND-2000, Thermo Fisher Scientific, Waltham, MA, USA). After extraction, RNA was stored at −80 °C.

### 4.4. RNA-Seq Analysis

Transcriptome analyses of Ganmi were performed by RNA sequencing, with plants of Newhall navel orange serving as the control. Pulps at 180 and 200 DAF were sampled for RNA-Seq analysis. The total RNAs were extracted. The library construction and sequencing were performed using the Illumina Hiseq (Illumina, San Diego, CA, USA). RNA sequencing was analyzed as described in a previous report [6]. The results of sequencing and the related quality controls are summarized in Table S9. The reference genome for this transcriptome sequencing was *Citrus sinensis* v3.0 (http://citrus.hzau.edu.cn/, accessed on 15 December 2025). Identification of differentially expressed genes (DEGs) (|log2 (Fold change)| ≥ 1, *p*-value < 0.05) was performed using DESeq2 (v1.26.0). Functional annotation of the DEGs was assigned using the Swiss-Prot, Pfam, KEGG, and GO databases.

### 4.5. WGCNA

Transcriptome data for 12 samples were normalized to fragments per kilobase of transcript per million mapped reads (FPKM) to select genes for gene co-expression network analysis of *C. sinensis*. Co-expression-network analysis was conducted with the WGCNA package in R software (version 4.1.1) with default parameters and minimum network size set to 20, as previously described [6,22]. After the network had been generated, key modules that might be related to fruit sugar accumulation in *C. sinensis* were identified according to correlations between modules and TSS. Functional annotation of genes within the module of interest was conducted by searching against the KEGG databases, and the network was mapped with Cytoscape v3.8.0 (http://apps.cytoscape.org/apps/cytohubba, accessed on 15 December 2025) [39,40]. Functional annotation of the genes in brown1 module was assigned using the Swiss-Prot, Pfam, KEGG, and GO databases.

### 4.6. Expression Analysis of the Candidate Key Genes

The RNA-seq data of orange fruit flesh from the twenty-first century at young fruit, fruit-coloring onset, and the fruit delayed-harvest stage were downloaded from the NCBI sequence read archive (SRA) with accession code PRJNA387319. RNA sequencing was analyzed as described in a previous report [6]. The reference genome for this transcriptome sequencing was *Citrus sinensis* v3.0 (http://citrus.hzau.edu.cn/, accessed on 15 December 2025). Fragments per kilobase of exon model per million mapped reads (FPKMs) were used to measure the transcript abundance of the genes.

## Figures and Tables

**Table 1:** Candidate transcription factors related to fruit sugar accumulation in pulps of navel orange.

ID	Annotation	GS	MM
*Cs_ont_2g004470*	NAC domain-containing protein 73; NAC73	0.895	−0.908
*Cs_ont_7g025420*	MYB1R1	0.840	−0.877
*Cs_ont_2g025530*	ERF113	−0.810	0.882
*Cs_ont_2g014800*	Heat stress transcription factor A-4; Hsf A-4	−0.884	0.907
*Cs_ont_9g006370*	Heat stress transcription factor A-6b; Hsf A-6b	−0.818	0.925
*Cs_ont_5g050360*	MYC2	−0.916	0.929
*Cs_ont_3g002820*	B-box zinc finger protein 21; BBX21	−0.923	0.937

## Data Availability

RNA-seq data of this study have been uploaded to the National Biotechnology Information Center (NCBI) SRA database with the primary accession code PRJNA1332904.

## Supplementary Materials

The following supporting information can be downloaded at https://www.mdpi.com/article/10.3390/ijms262412161/s1.
