# Supplementary material for: Identification of Novel Regulators of Fruit Sugar Accumulation Based on Transcriptome and WGCNA in Citrus sinensis
Source: Int J Mol Sci. 2025 Dec 18;26(24):12161. doi: 10.3390/ijms262412161 (PMC12733902; doi:10.3390/ijms262412161)
Supplement: Supplementary file 1 [file ijms-26-12161-s001.zip › captions.pdf]

Figure S1: TSS of fruit pulps of Ganmi and NHE navel orange at mature stage. * and ** denote significant difference at the 0.05 and 0.01 probability levels, respectively.

Figure S2: Identification of DEGs related to fruit ripening. (A-B) are volcano plot of differentially expressed genes in Ganmi and Newhall navel orange at 180, and 200 DAF respectively.

Figure S3: GO enrichment analyses for DEGs. (A-B) are 20 GO biological process terms of differentially expressed genes in Ganmi and Newhall navel orange at 180, and 200 DAF respectively.

Figure S4: Number of genes in each module.

Table S1: Identification of differentially expressed genes related to sugar accumulation between GM and Newhall navel orange.

Table S2: Identification and functional annotation of differentially expressed genes in pulps between GM and and Newhall navel orange both at 180 and 200 DAF.

Table S3: KEGG enrichment analysis of all DEGs in pulps of Ganmi navel orange at 180 DAF.

Table S4: KEGG enrichment analysis of all DEGs in pulps of Ganmi navel orange at 200 DAF.

Table S5: Biological_process (GO) enrichment analysis of DEGs in fruit of Ganmi navel orange at 180 DAF.

Table S6: Biological_process (GO) enrichment analysis of DEGs in fruit of Ganmi navel orange at 200 DAF.

Table S7: Genes expression and functional annotation of genes in brown1 module.

Table S8: Expression characterization of seven genes in fruits at different developmental stages.

Table S9: Summary of data from RNA sequencing.
