# Supplementary figures and images for: Identification of Novel Regulators of Fruit Sugar Accumulation Based on Transcriptome and WGCNA in Citrus sinensis
Source: Int J Mol Sci. 2025 Dec 18;26(24):12161. doi: 10.3390/ijms262412161 (PMC12733902; doi:10.3390/ijms262412161)

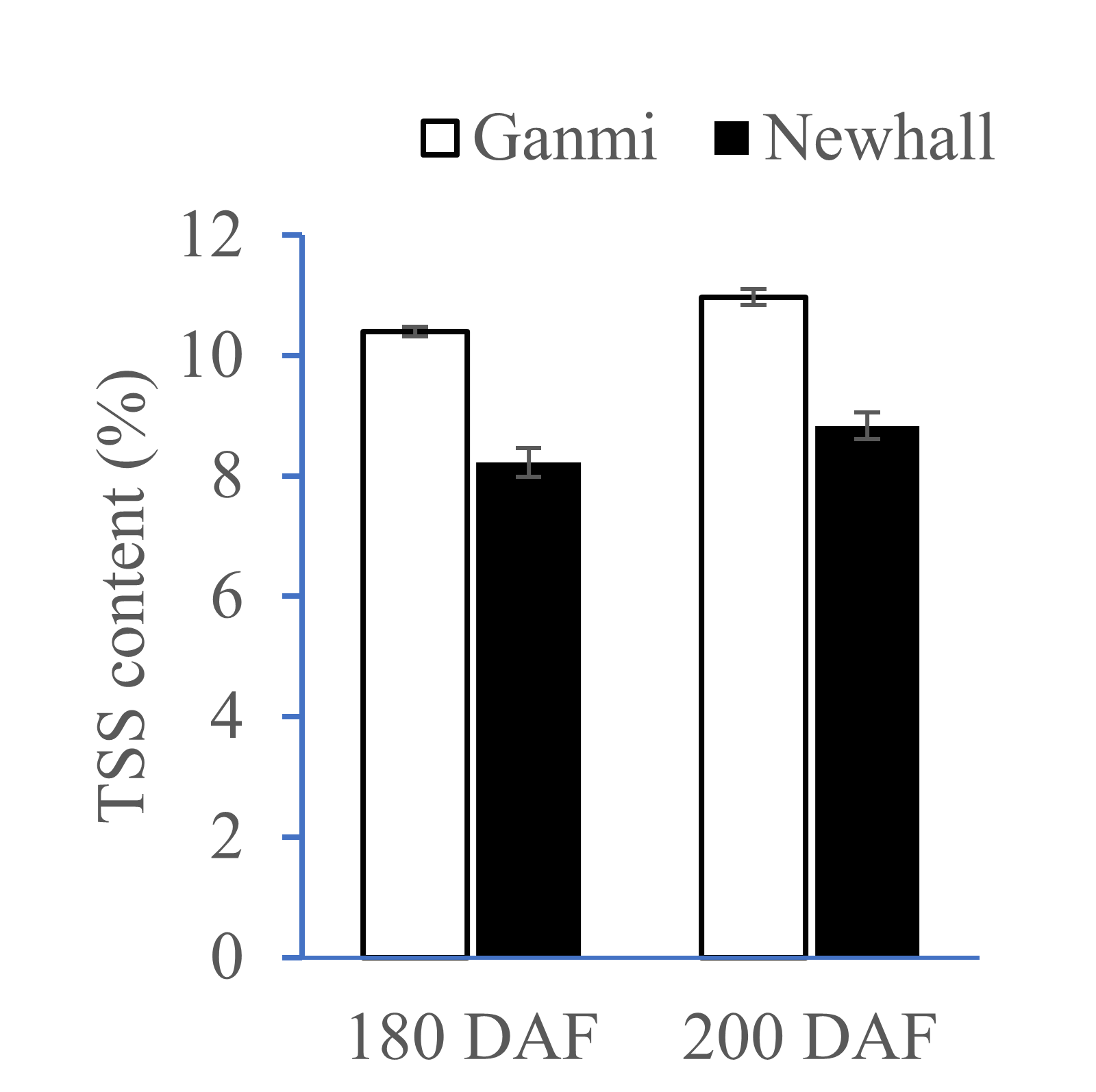

Supplement: Supplementary file 1 [file ijms-26-12161-s001.zip › Figure S1.TIF]

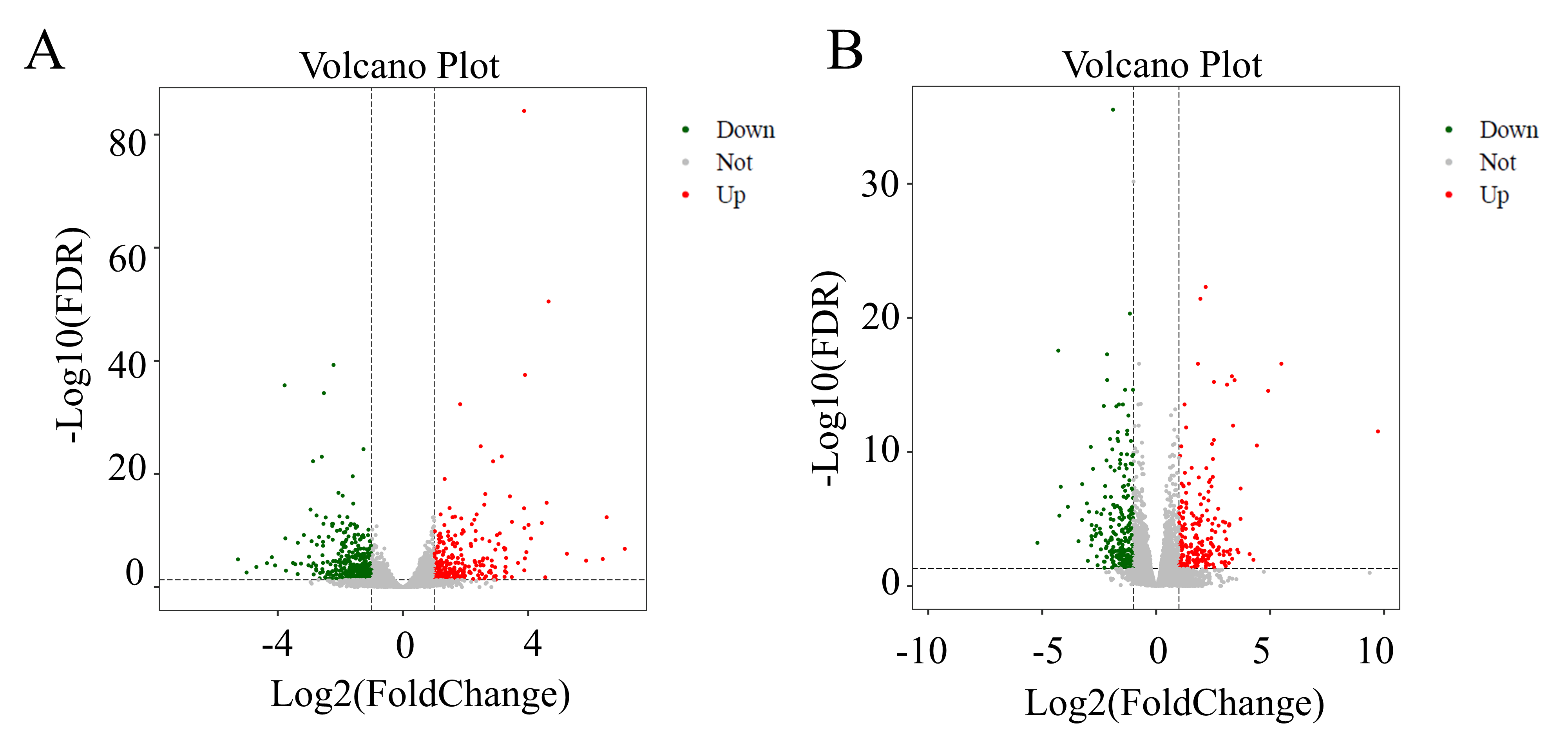

Supplement: Supplementary file 1 [file ijms-26-12161-s001.zip › Figure S2.TIF]

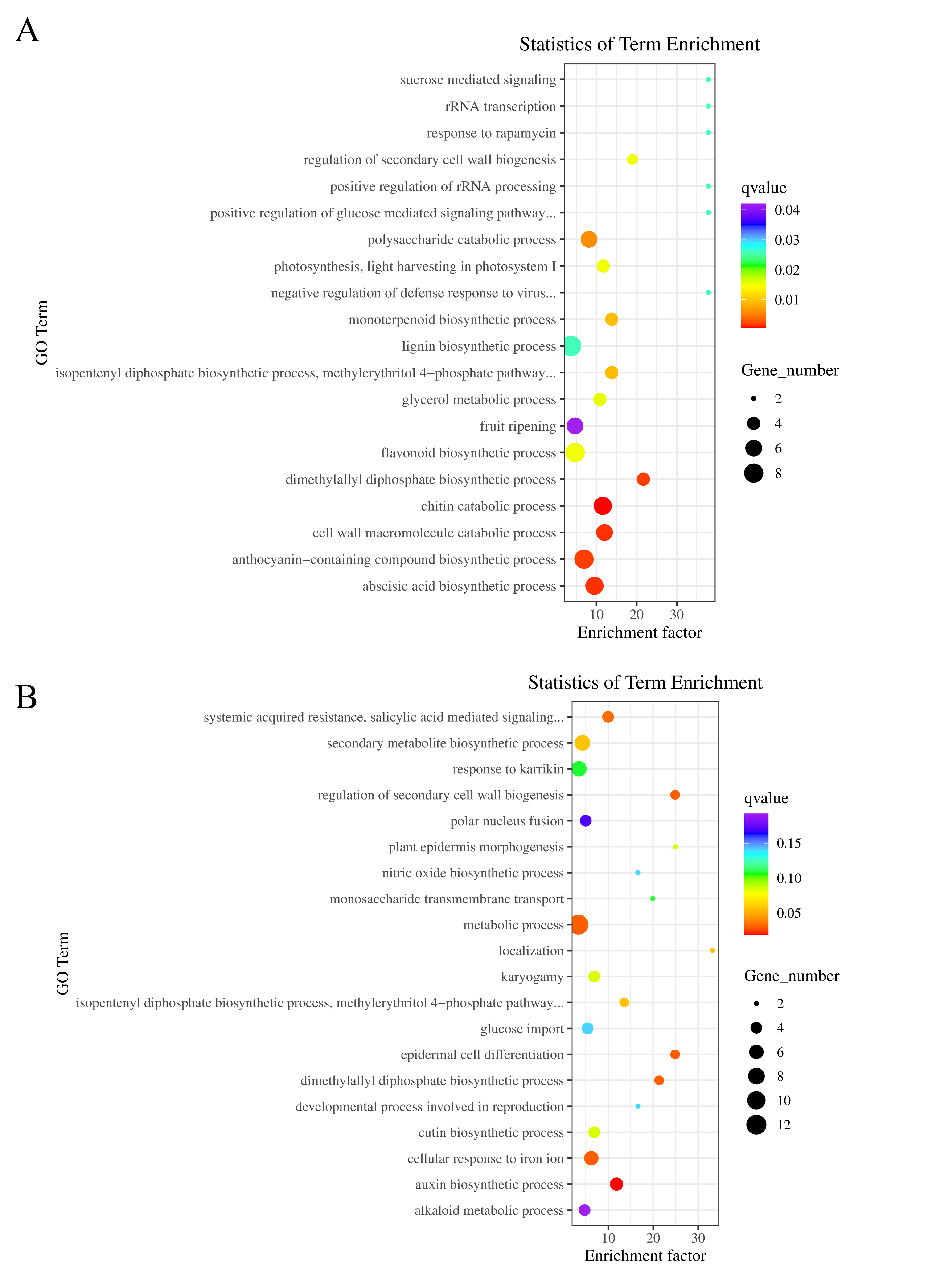

Supplement: Supplementary file 1 [file ijms-26-12161-s001.zip › Figure S3.TIF]

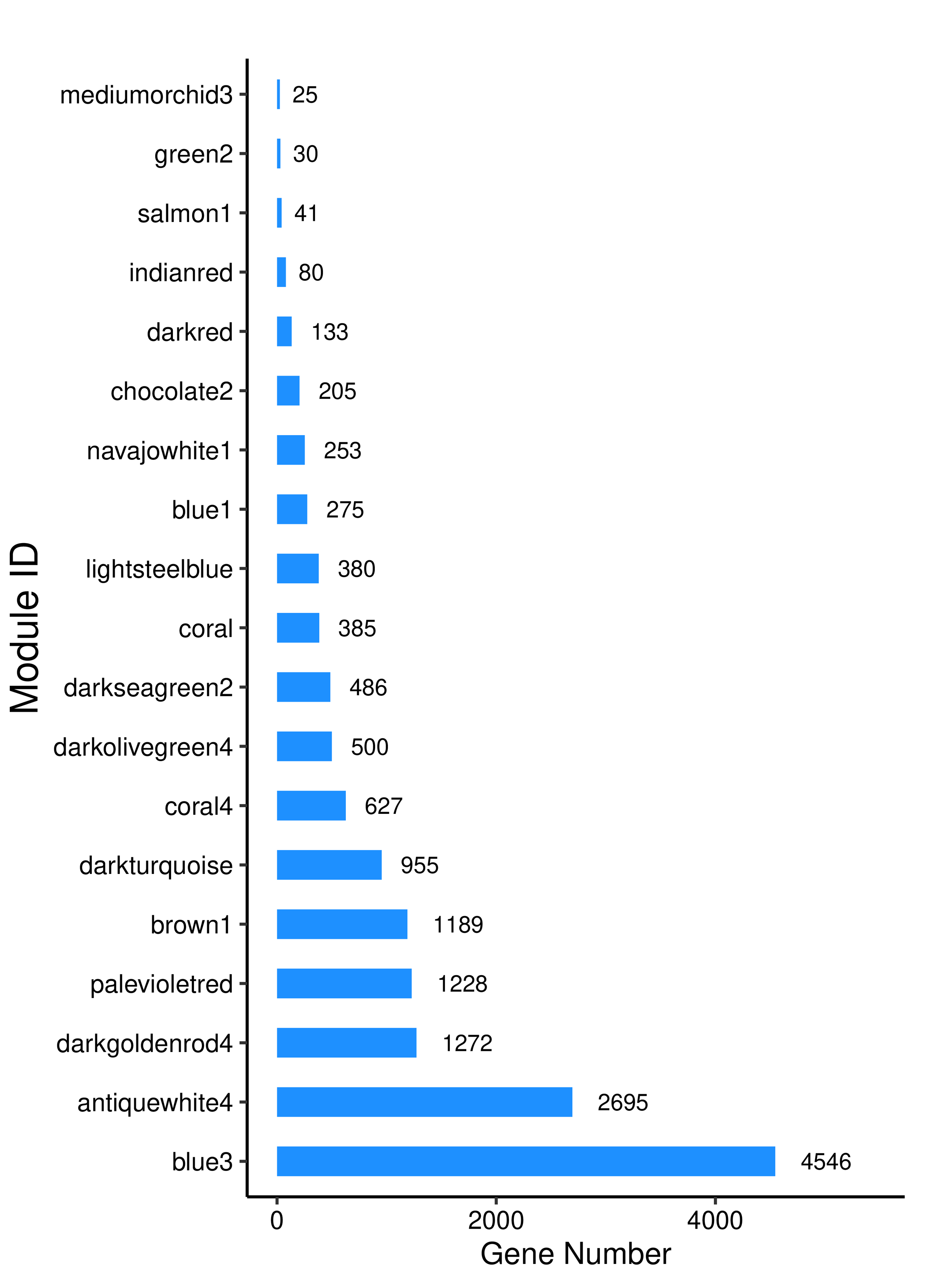

Supplement: Supplementary file 1 [file ijms-26-12161-s001.zip › Figure S4.TIF]
